# Supplementary material for: Hatsusamides A and B: Two New Metabolites Produced by the Deep-Sea-Derived Fungal Strain Penicillium steckii FKJ-0213
Source: Mar Drugs. 2020 Oct 12;18(10):513. doi: 10.3390/md18100513 (PMC7601204; doi:10.3390/md18100513)
Supplement: Supplementary file 1 [file marinedrugs-18-00513-s001.pdf]

marine drugs

MDPI

Supplemetary Material

Hatsusamides A and B: Two New Metabolites Produced by the Deep-Sea-Derived Fungal Strain *Penicillium steckii* FKJ-0213

Hirotaka Matsuo [1,2,3,*], Rei Hokari [1], Aki Ishiyama [1,2], Masato Iwatsuki [1,2], Mayuka Higo [1], Kenichi Nonaka [1,2], Yuriko Nagano [4], Yōko Takahashi [1], Satoshi Ōmura [1] and Takuji Nakashima [1,2,5,*]

[1] Ōmura Satoshi Memorial Institute, Kitasato University, 5-9-1 Shirokane, Minatok-ku, Tokyo 108-8641, Japan; hokari@lisci.kitasato-u.ac.jp (R.H.); ishiyama@lisci.kitasato-u.ac.jp (A.I.); iwatsuki@lisci.kitasato-u.ac.jp (M.I.); mayuka@lisci.kitasato-u.ac.jp (M.H.); ken@lisci.kitasato-u.ac.jp (K.N.); ytakaha@lisci.kitasato-u.ac.jp (Y.T.); omuras@insti.kitasato-u.ac.jp (S.Ō.)
[2] Department of Drug Discover Sciences, Graduate School of Infection Control Sciences, 5-9-1 Shirokane, Minatok-ku, Tokyo 108-8641, Japan
[3] Research Center for Medicinal Plant Resources, National Institutes of Biomedical Innovation, Health and Nutrition, 1-2 Hachimandai, Tsukuba, Ibaraki 305-8043, Japan
[4] Department of Marine Biodiversity Research, Japan Agency for Marine-Earth Science and Technology, 2-15 Natsushima-cho, Yokosuka, Kanagawa 237-0061, Japan; y.nagano@jamstec.go.jp
[5] Research Innovation Center, Waseda University, 513 Waseda tsurumakicho, Shinjuku-ku, Tokyo 162-0041, Japan
* Correspondence: matsu-h@lisci.kitasato-u.ac.jp (H.M.); takuji@lisci.kitasato-u.ac.jp (T.N.); Tel./Fax: +81-3-5791-6450 (H.M.)

List of supporting information:

Figure S1. ^{1}H NMR (500 MHz, acetone-d_6) spectrum of **1**

Figure S2. ^{13}C NMR (120 MHz, acetone-d_6) spectrum of **1**

Figure S3. ^{1}H-^{1}H COSY (500 MHz, acetone-d_6) spectrum of **1**

Figure S4. HMQC (500 MHz, acetone-d_6) spectrum of **1**

Figure S5. HMBC (500 MHz, acetone-d_6) spectrum of **1**

Figure S6. Total ion current (TIC) chromatogram of hydrolysate of **1**

Figure S7. Comparison of ^{1}H-NMR of isolated tanzawaic acid B and hydrolysated tanzawaic acid B derived from **1**.

Figure S8. Comparison of ^{1}H-NMR of isolated trichodermamide C and hydrolysated trichodermamide C derived from **1**.

Figure S9. Single-crystal X-ray crystallographic data of **3**

Figure S10. ^{1}H NMR (500 MHz, acetone-d_6) spectrum of **2**

Figure S11. ^{13}C NMR (120 MHz, acetone-d_6) spectrum of **2**

Figure S12. ^{1}H-^{1}H COSY (500 MHz, acetone-d_6) spectrum of **2**

Figure S13. HMQC (500 MHz, acetone-d_6) spectrum of **2**

Figure S14. HMBC (500 MHz, acetone-d_6) spectrum of **2**

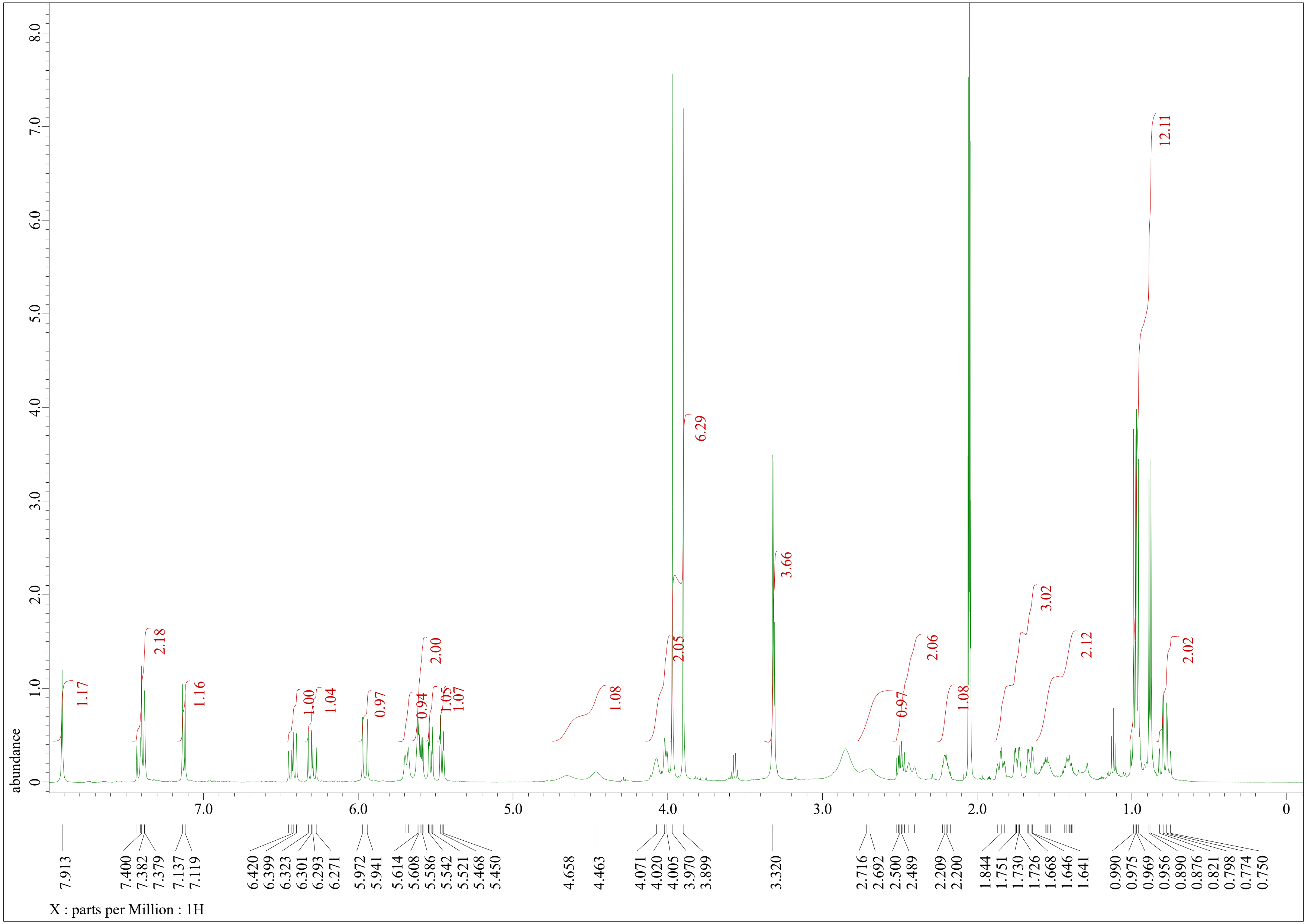
abundance
X : parts per Million : 1H
1.17
2.18
1.16
1.00
1.04
0.97
0.94
2.00
1.05
1.07
1.08
2.05
6.29
3.66
0.97
2.06
1.08
3.02
2.12
12.11
2.02
7.913
7.400
7.382
7.379
7.137
7.119
6.420
6.399
6.323
6.301
6.293
6.271
5.972
5.941
5.614
5.608
5.586
5.542
5.521
5.468
5.450
4.658
4.463
4.071
4.020
4.005
3.970
3.899
3.320
2.716
2.692
2.500
2.489
2.209
2.200
1.844
1.751
1.730
1.726
1.668
1.646
1.641
0.990
0.975
0.969
0.956
0.890
0.876
0.821
0.798
0.774
0.750

Figure S1. ^{1}H NMR (500 MHz, acetone-d_6) spectrum of **1**

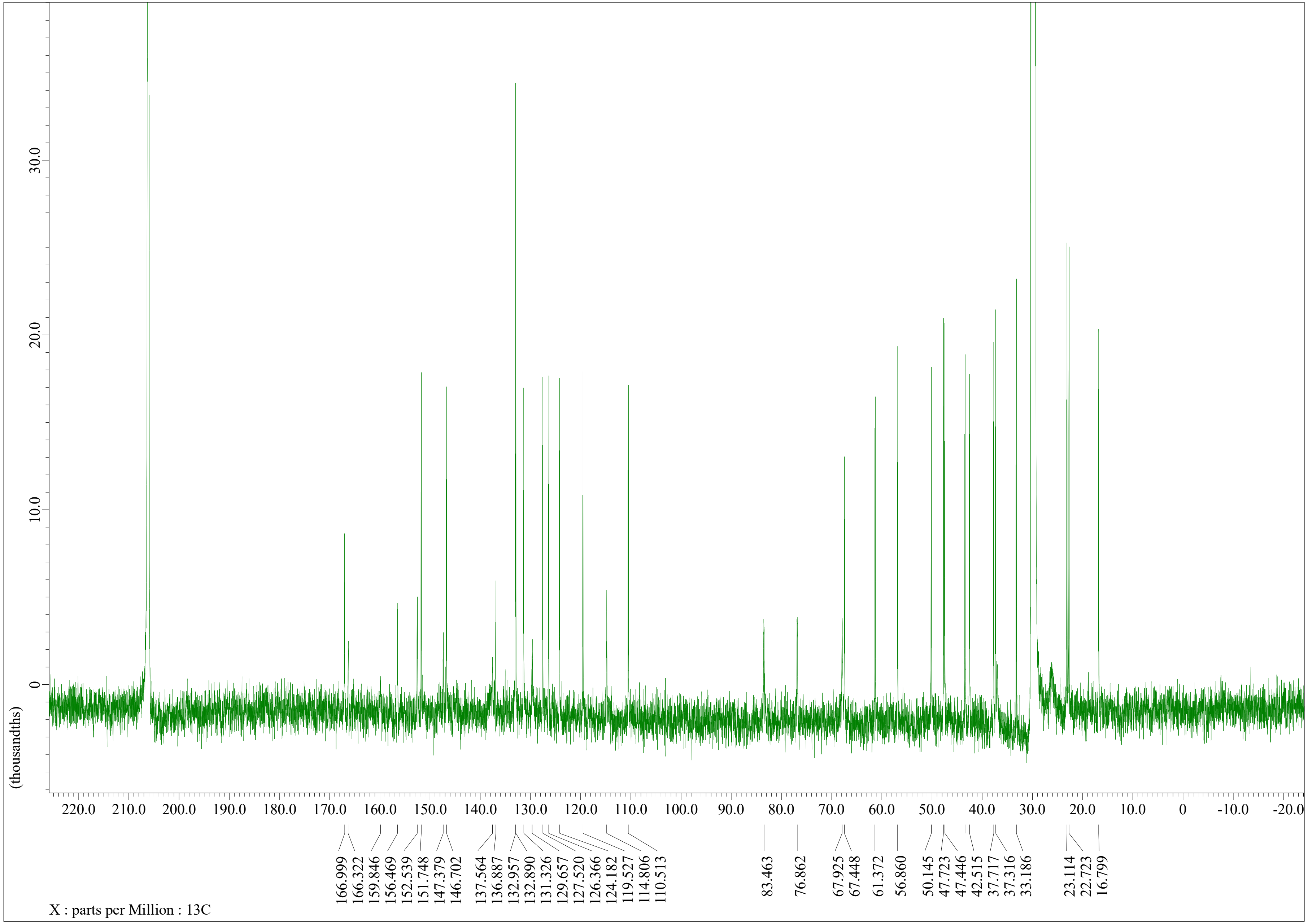
(thousandths)
30.0
20.0
10.0
0
220.0 210.0 200.0 190.0 180.0 170.0 160.0 150.0 140.0 130.0 120.0 110.0 100.0 90.0 80.0 70.0 60.0 50.0 40.0 30.0 20.0 10.0 0 -10.0 -20.0
166.999
166.322
159.846
156.469
152.539
151.748
147.379
146.702
137.564
136.887
132.957
132.890
131.326
129.657
127.520
126.366
124.182
119.527
114.806
110.513
83.463
76.862
67.925
67.448
61.372
56.860
50.145
47.723
47.446
42.515
37.717
37.316
33.186
23.114
22.723
16.799
X : parts per Million : 13C

Figure S2. ^{13}C NMR (125 MHz, acetone-d_6) spectrum of **1**

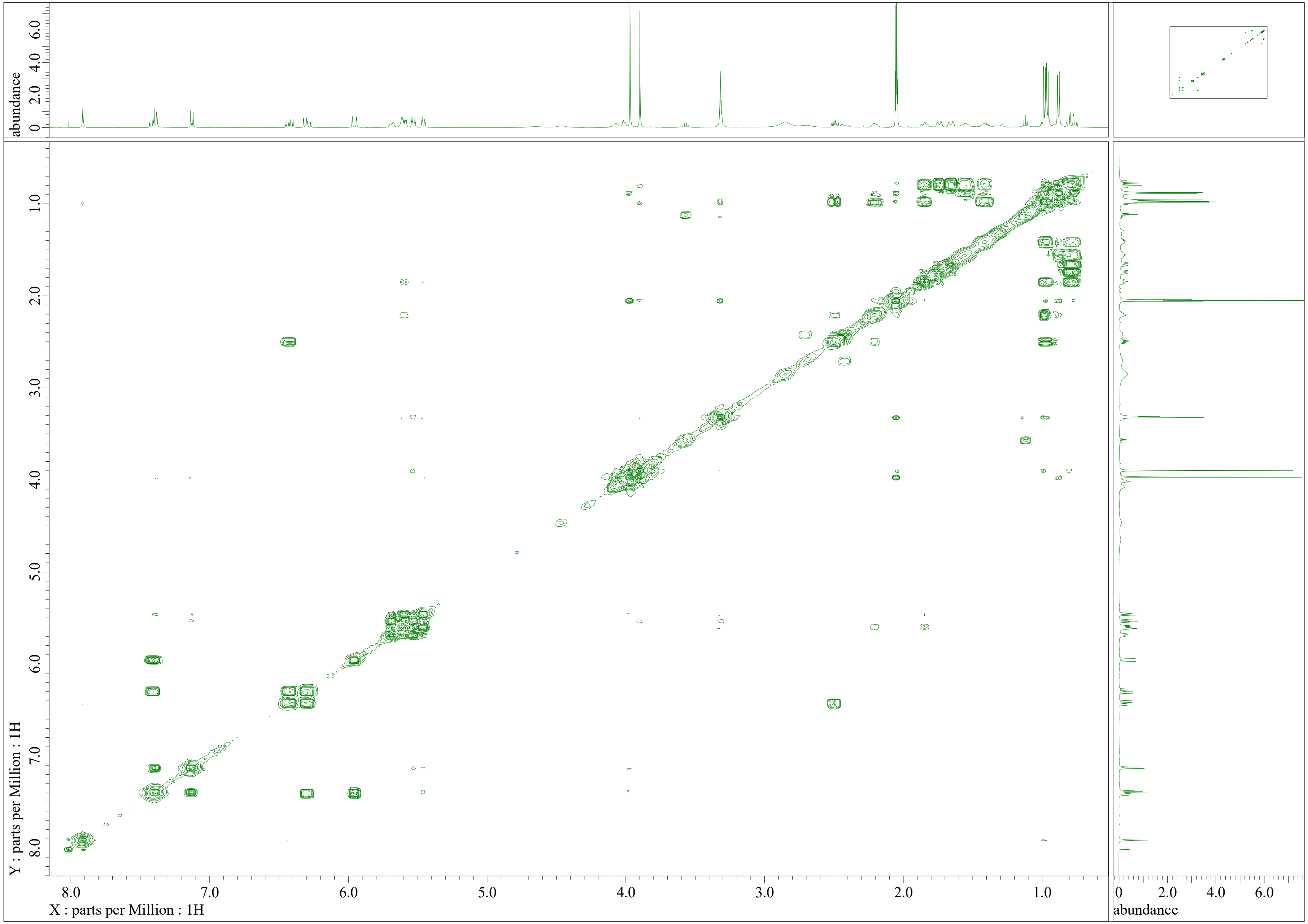
abundance
0
2.0
4.0
6.0
Y : parts per Million : 1H
1.0
2.0
3.0
4.0
5.0
6.0
7.0
8.0
8.0
7.0
6.0
5.0
4.0
3.0
2.0
1.0
X : parts per Million : 1H
0
2.0
4.0
6.0
abundance

Figure S3. ^{1}H-^{1}H COSY (500 MHz, acetone-d_6) spectrum of **1**

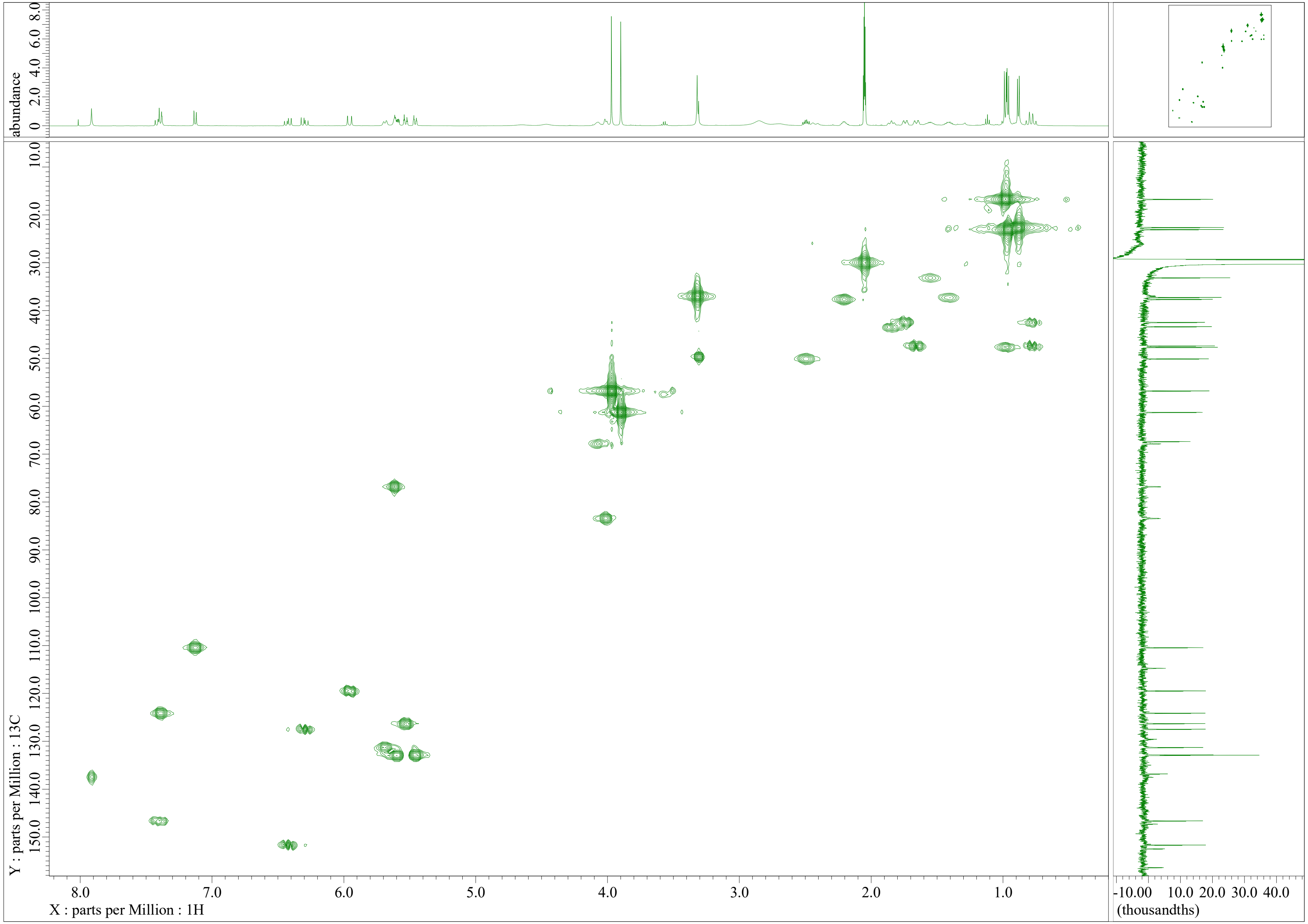
abundance
Y : parts per Million : 13C
X : parts per Million : 1H
(thousandths)

Figure S4. HMQC (500 MHz, acetone-d_6) spectrum of **1**

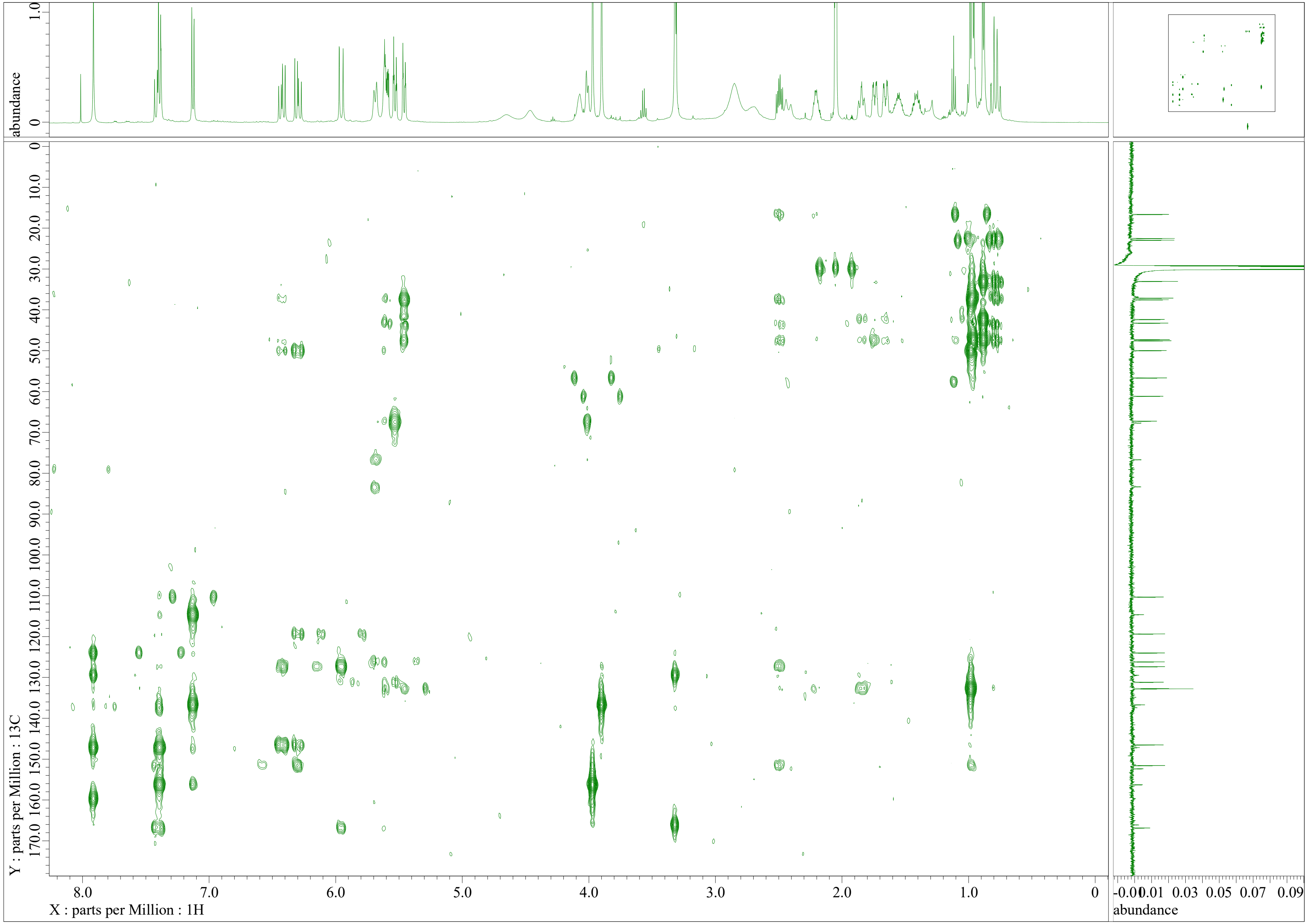
abundance
Y : parts per Million : 13C
X : parts per Million : 1H
abundance

Figure S5. HMBC (500 MHz, acetone-d_6) spectrum of **1**

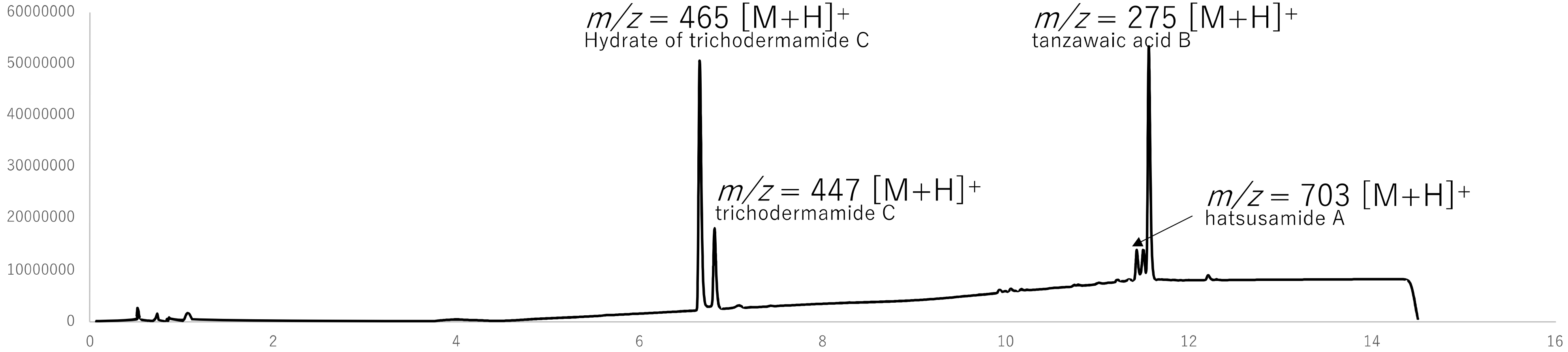

Figure S6. Total ion current (TIC) chromatogram of hydrolysate of **1**

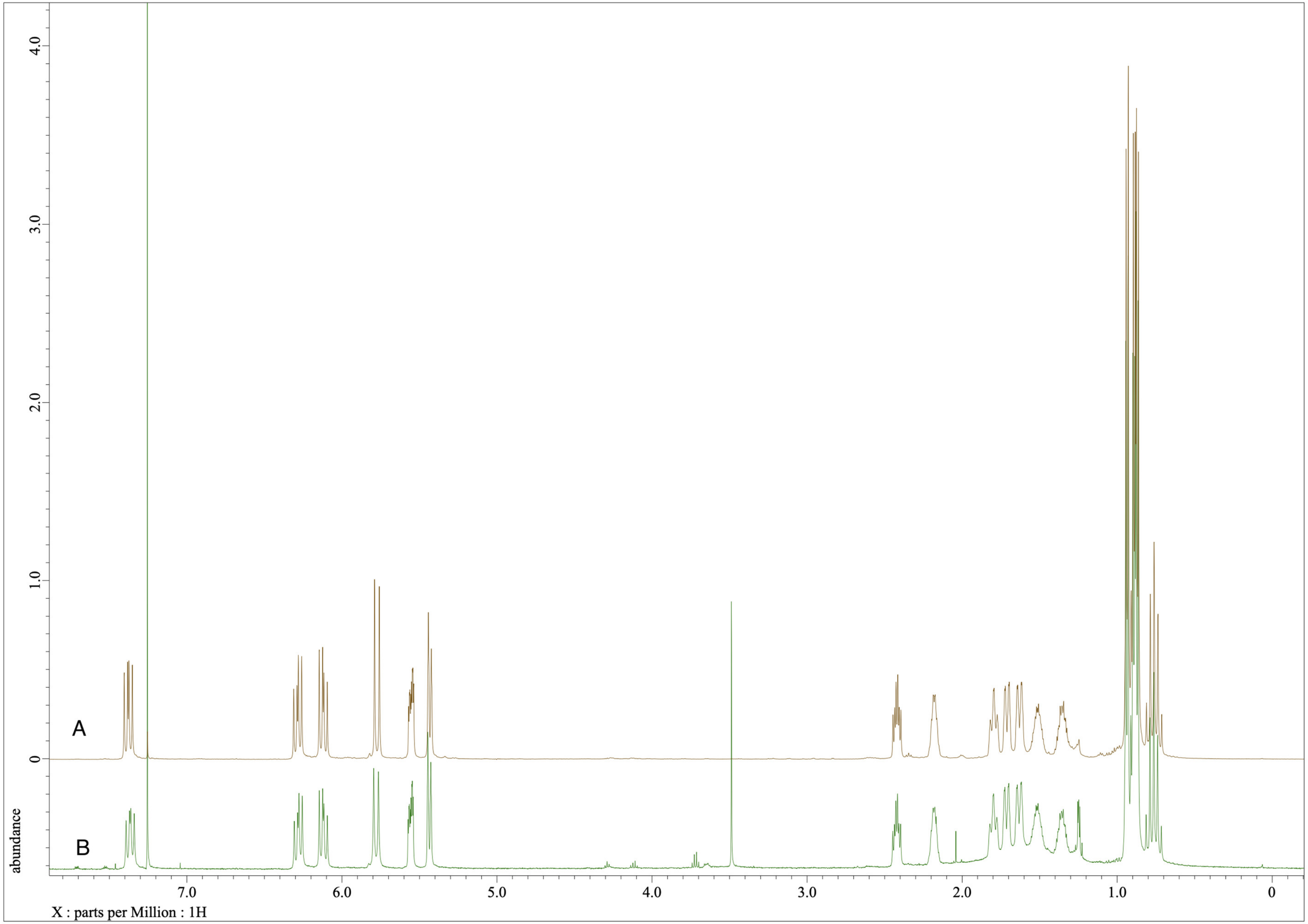
4.0
3.0
2.0
1.0
0
abundance
A
B
7.0
6.0
5.0
4.0
3.0
2.0
1.0
0
X : parts per Million : 1H

Figure S7. Comparison of ^{1}H-NMR (in $CDCl_3$) of isolated tanzawaic acid B (A: brown) and hydrolysated tanzawaic acid B (B: green) derived from **1**.

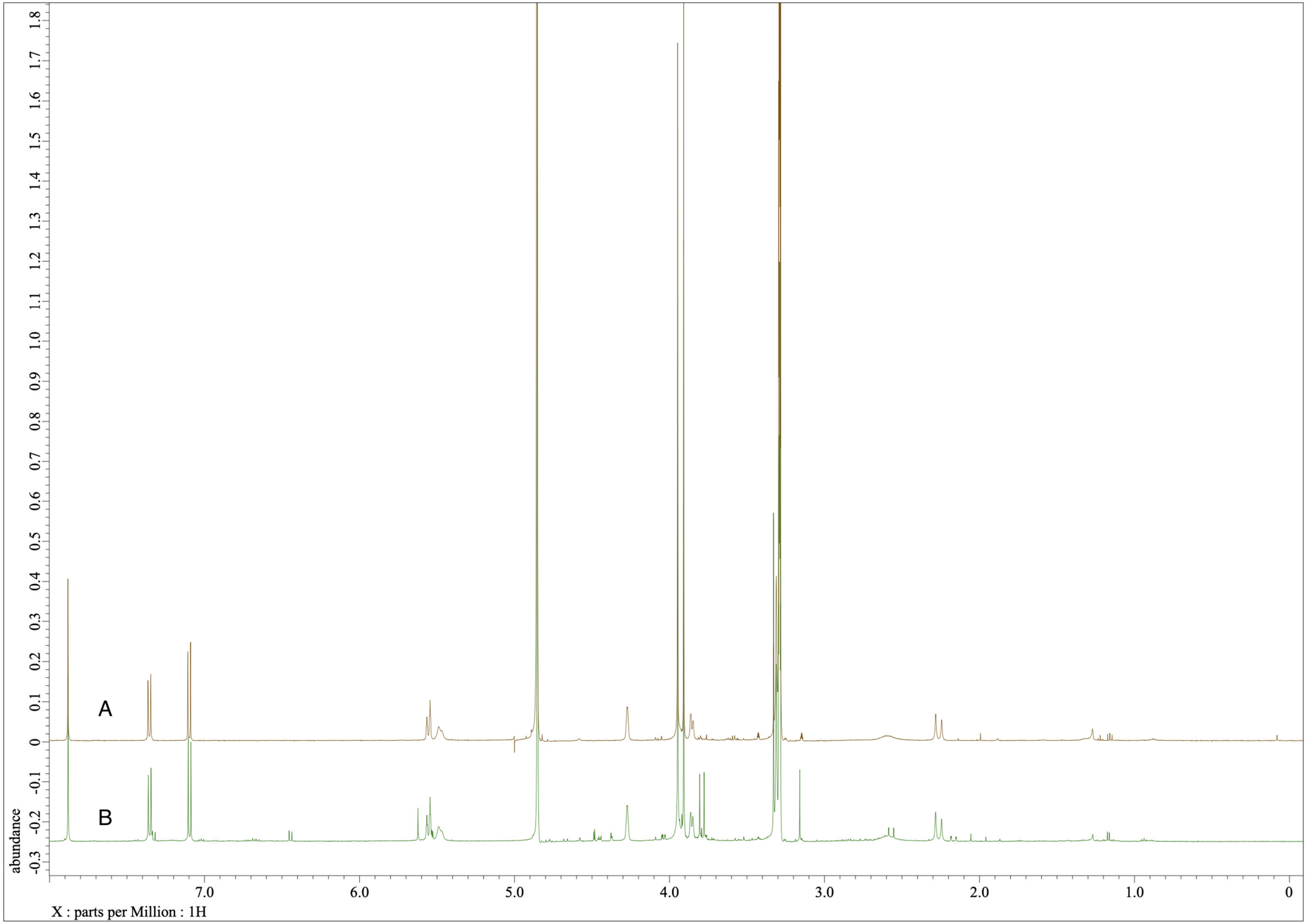
abundance
A
B
X : parts per Million : 1H
7.0
6.0
5.0
4.0
3.0
2.0
1.0
0

Figure S8. Comparison of ^{1}H-NMR (in methanol-d_4) of isolated trichodermamide C (A: brown) and hydrolysated trichodermamide C (B: green) derived from **1**.

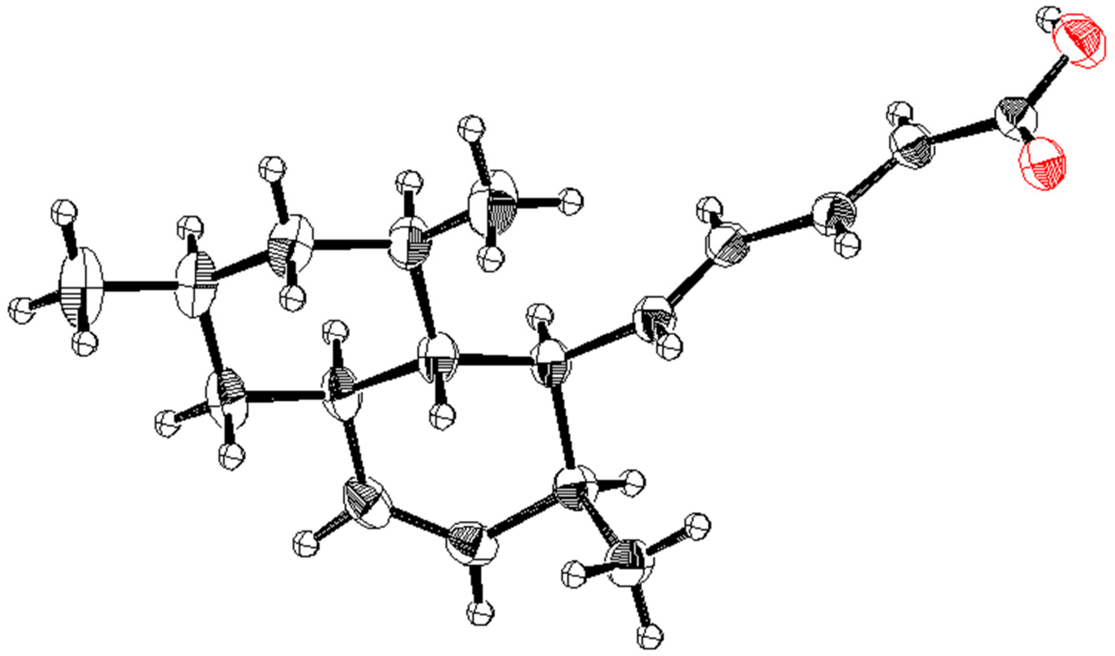

Figure S9. Single-crystal X-ray crystallographic data of **3**

The crystal of **3**, $C_{18}H_{26}O_2$ as the space group $P2_12_12_1$ (#19) with a= 7.27873(13) Å, b= 12.7268(2) Å, c= 36.8385(7) Å, V=3412.53(11) Å^3, Z= 8, D_{calcd}= 1.068 g/cm^3, μ= 5.268 cm^{-1} and T= 23.0°C. X-ray intensity data were collected on a Rigaku R-AXIS RAPID diffractometer employing graphite-monochromated Cu Kα radiation (λ=1.54187 Å) and the ω scan technique. The structure was solved by direct methods. For refinement, 6238 unique reflections with F^2>2.0σ(F^2) were used. Full-matrix least-squares refinement based on F^2, minimizing the quantity $\Sigma w(Fo^2 - F^2)^2$ with w= $1/[\sigma^2 (Fo^2)+(0.0648P)^2+0.9177P]$ where P= $(Max(Fo^2,0)+2Fc^2)/3$, GOF= 1.076, R1=0.0715, Rw=0.1890 and flack parameter=-0.05(9).

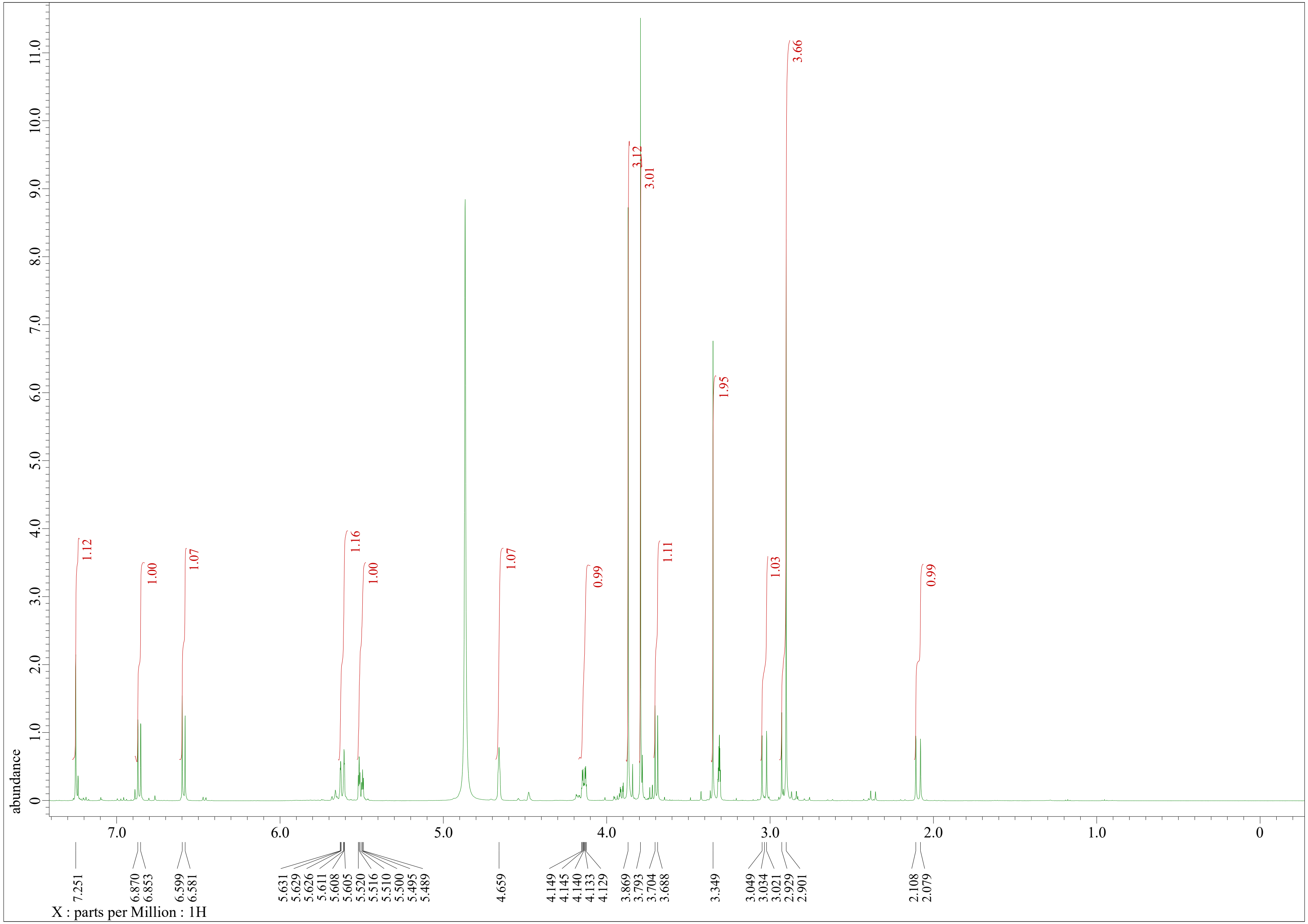
abundance
0
1.0
2.0
3.0
4.0
5.0
6.0
7.0
8.0
9.0
10.0
11.0
7.0
6.0
5.0
4.0
3.0
2.0
1.0
0
1.12
1.00
1.07
1.16
1.00
1.07
0.99
3.12
3.01
1.11
1.95
1.03
3.66
0.99
7.251
6.870
6.853
6.599
6.581
5.631
5.629
5.626
5.611
5.608
5.605
5.520
5.516
5.510
5.500
5.495
5.489
4.659
4.149
4.145
4.140
4.133
4.129
3.869
3.793
3.704
3.688
3.349
3.049
3.034
3.021
2.929
2.901
2.108
2.079
X : parts per Million : 1H

Figure S10. ^{1}H NMR (500 MHz, methanol-d_4) spectrum of **2**

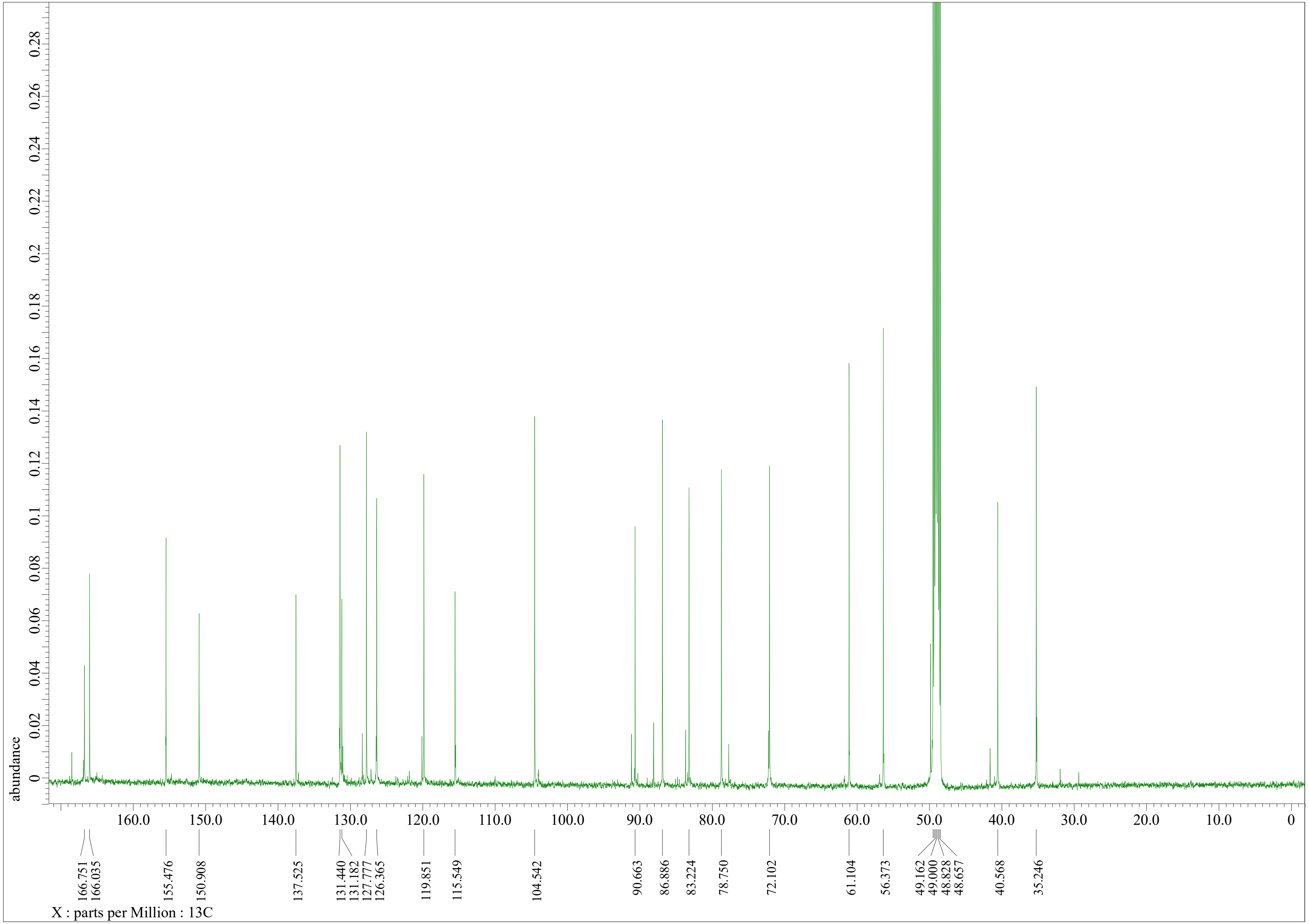

abundance
0
0.02
0.04
0.06
0.08
0.1
0.12
0.14
0.16
0.18
0.2
0.22
0.24
0.26
0.28
160.0
150.0
140.0
130.0
120.0
110.0
100.0
90.0
80.0
70.0
60.0
50.0
40.0
30.0
20.0
10.0
0
166.751
166.035
155.476
150.908
137.525
131.440
131.182
127.777
126.365
119.851
115.549
104.542
90.663
86.886
83.224
78.750
72.102
61.104
56.373
49.162
49.000
48.828
48.657
40.568
35.246
X : parts per Million : 13C

Figure S11. ^{13}C NMR (125 MHz, methanol-d_4) spectrum of **2**

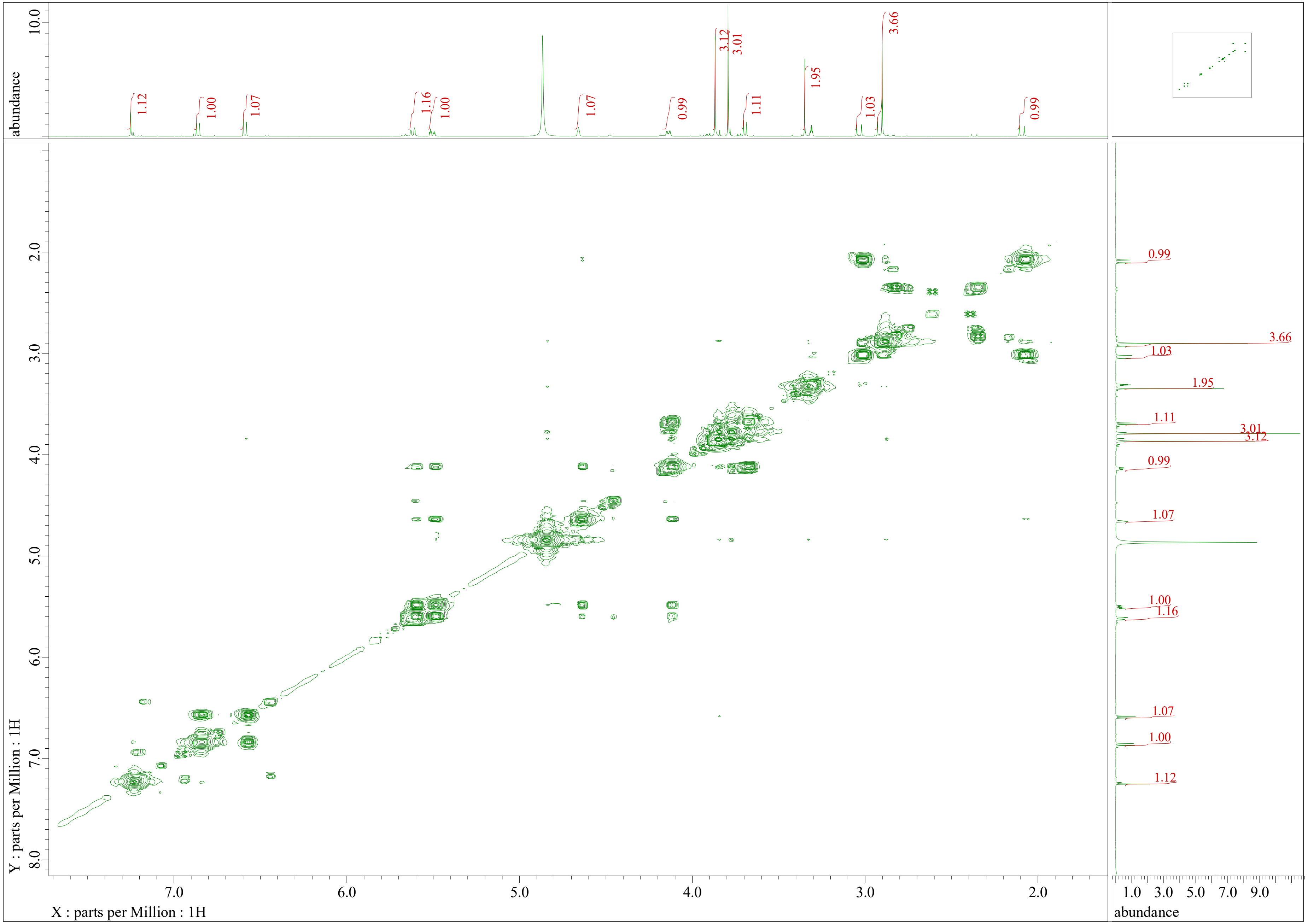

abundance
Y : parts per Million : 1H
X : parts per Million : 1H
abundance
1.12
1.00
1.07
1.16
1.00
1.07
0.99
3.12
3.01
1.11
1.95
1.03
3.66
0.99

Figure S12. ^{1}H-^{1}H COSY (500 MHz, methanol-d_4) spectrum of **2**

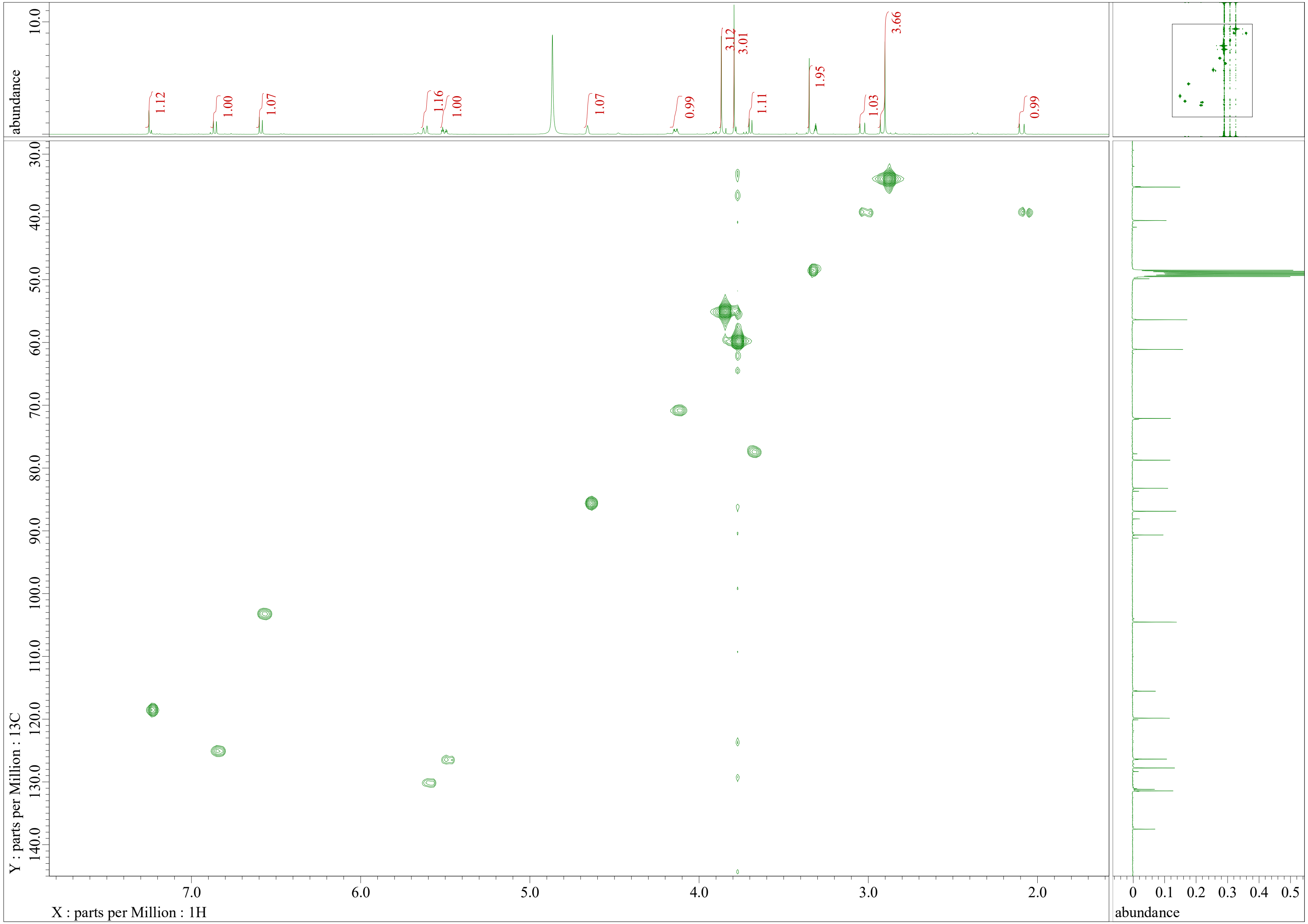
abundance
10.0
1.12
1.00
1.07
1.16
1.00
1.07
0.99
3.12
3.01
1.11
1.95
1.03
3.66
0.99
30.0
40.0
50.0
60.0
70.0
80.0
90.0
100.0
110.0
120.0
130.0
140.0
Y : parts per Million : 13C
7.0
6.0
5.0
4.0
3.0
2.0
X : parts per Million : 1H
0
0.1
0.2
0.3
0.4
0.5
abundance

Figure S13. HMQC (500 MHz, methanol-d_4) spectrum of **2**

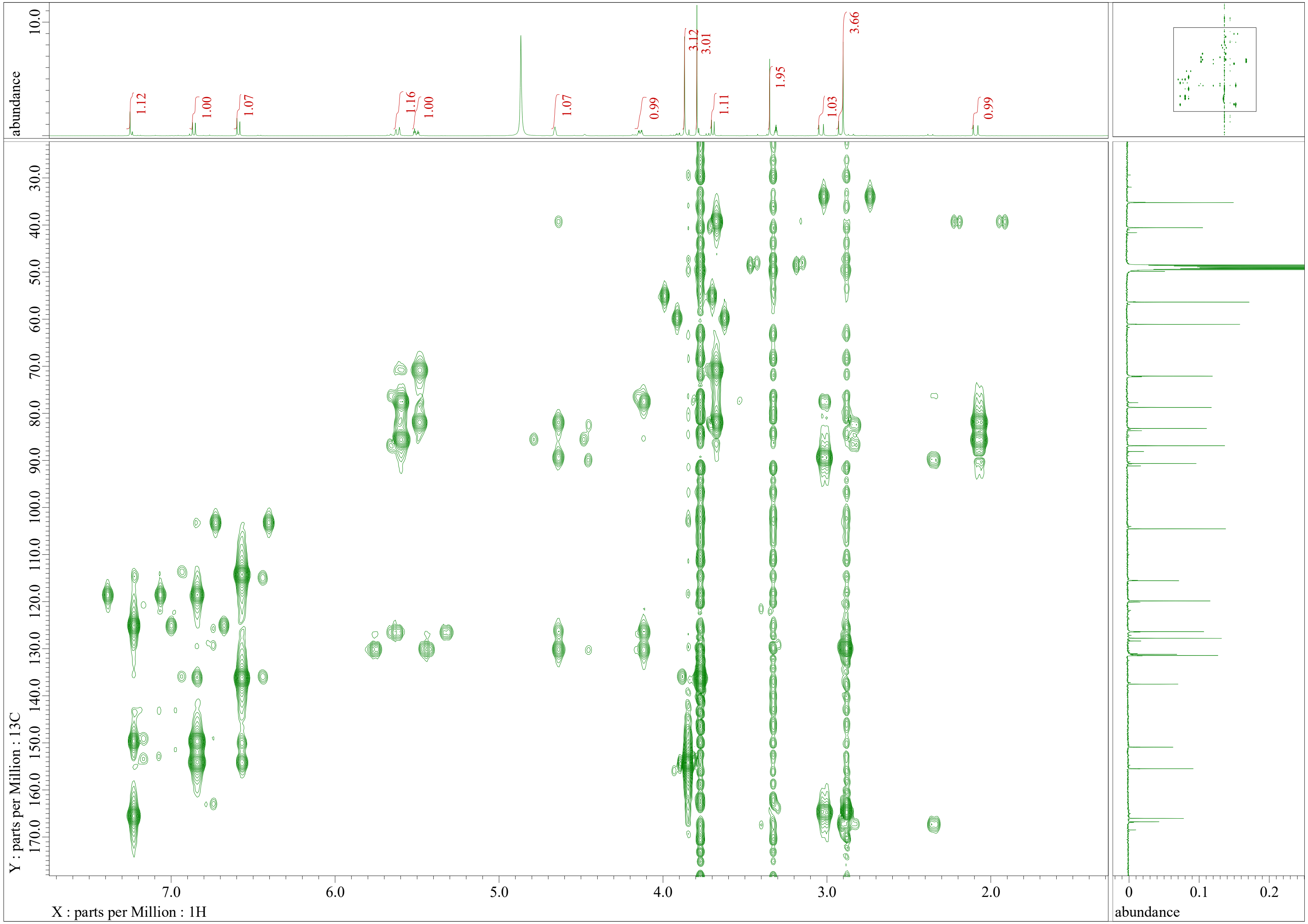
abundance
10.0
1.12
1.00
1.07
1.16
1.00
1.07
0.99
3.12
3.01
1.11
1.95
1.03
3.66
0.99
Y : parts per Million : 13C
30.0
40.0
50.0
60.0
70.0
80.0
90.0
100.0
110.0
120.0
130.0
140.0
150.0
160.0
170.0
7.0
6.0
5.0
4.0
3.0
2.0
X : parts per Million : 1H
0
0.1
0.2
abundance

Figure S14. HMBC (500 MHz, methanol-d_4) spectrum of **2**

© 2020 by the authors. Licensee MDPI, Basel, Switzerland. This article is an open access article distributed under the terms and conditions of the Creative Commons Attribution (CC BY) license (http://creativecommons.org/licenses/by/4.0/).
